# Supplementary material for: Bio-inspired creation of heterogeneous reaction vessels via polymerization of supramolecular ion pair
Source: Nat Commun. 2019 Jul 11;10:3059. doi: 10.1038/s41467-019-11080-5 (PMC6624306; doi:10.1038/s41467-019-11080-5)
Supplement: Supplementary file 1 — Supplementary Information [file 41467_2019_11080_MOESM1_ESM.docx]

**Supplementary Information**

**Bio-Inspired Creation of Heterogeneous Reaction Vessels via Polymerization of Supramolecular Ion Pairs**

Dong et al.

**Supplementary Figures**


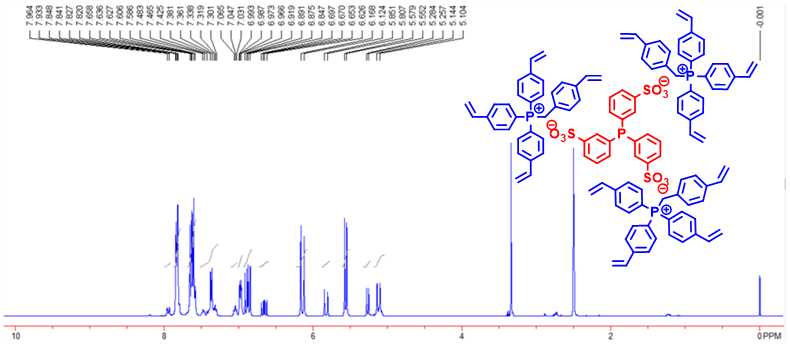

**
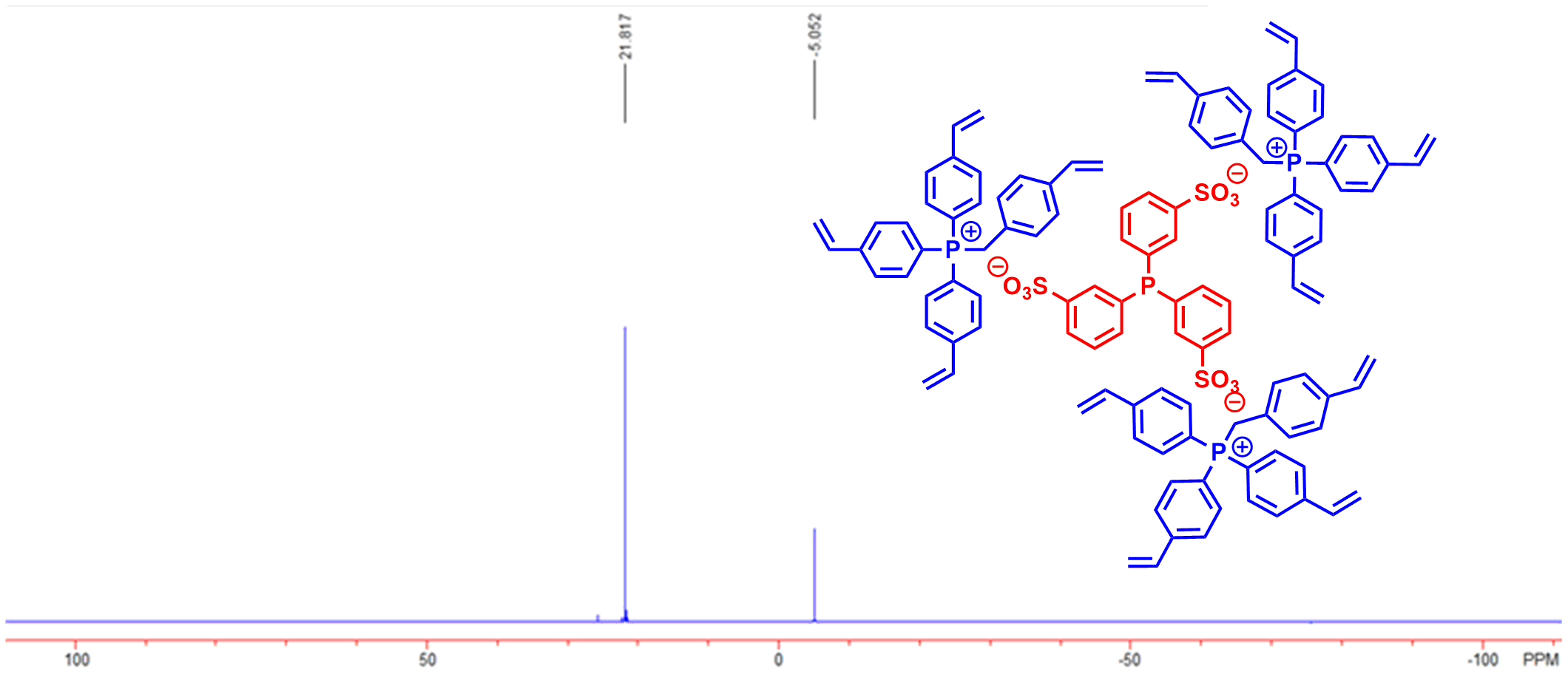
**

**Supplementary Figure 1 | ^1^H, ^13^C, and ^31^P NMR spectra of VSA-PPh_3_.**

**Supplementary Figure 2 | ESI-TOF-MS spectra of VSA-PPh_3_.** Above the dashed line is the cationic part detected under the positive ion mode and below the dashed line is the anionic part detected under the negative ion mode.

**Supplementary Figure 3 | ^1^H, ^13^C, and ^31^P NMR spectra of VSA-Xantphos.**

**Supplementary Figure 4 |** **ESI-TOF-MS spectra of VSA-Xantphos.** Above the dashed line is the cationic part detected under the positive ion mode and below the dashed line is the anionic part detected under the negative ion mode.

**
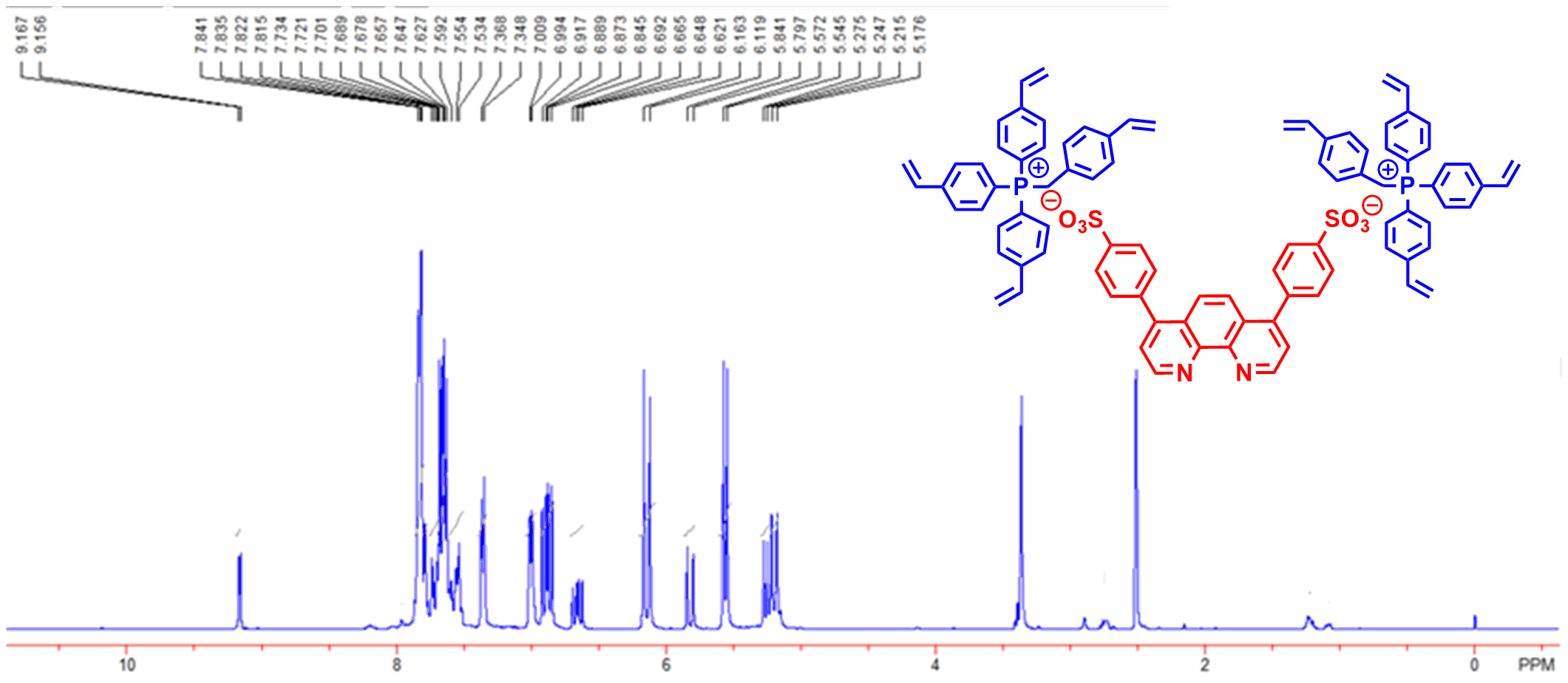
**

**
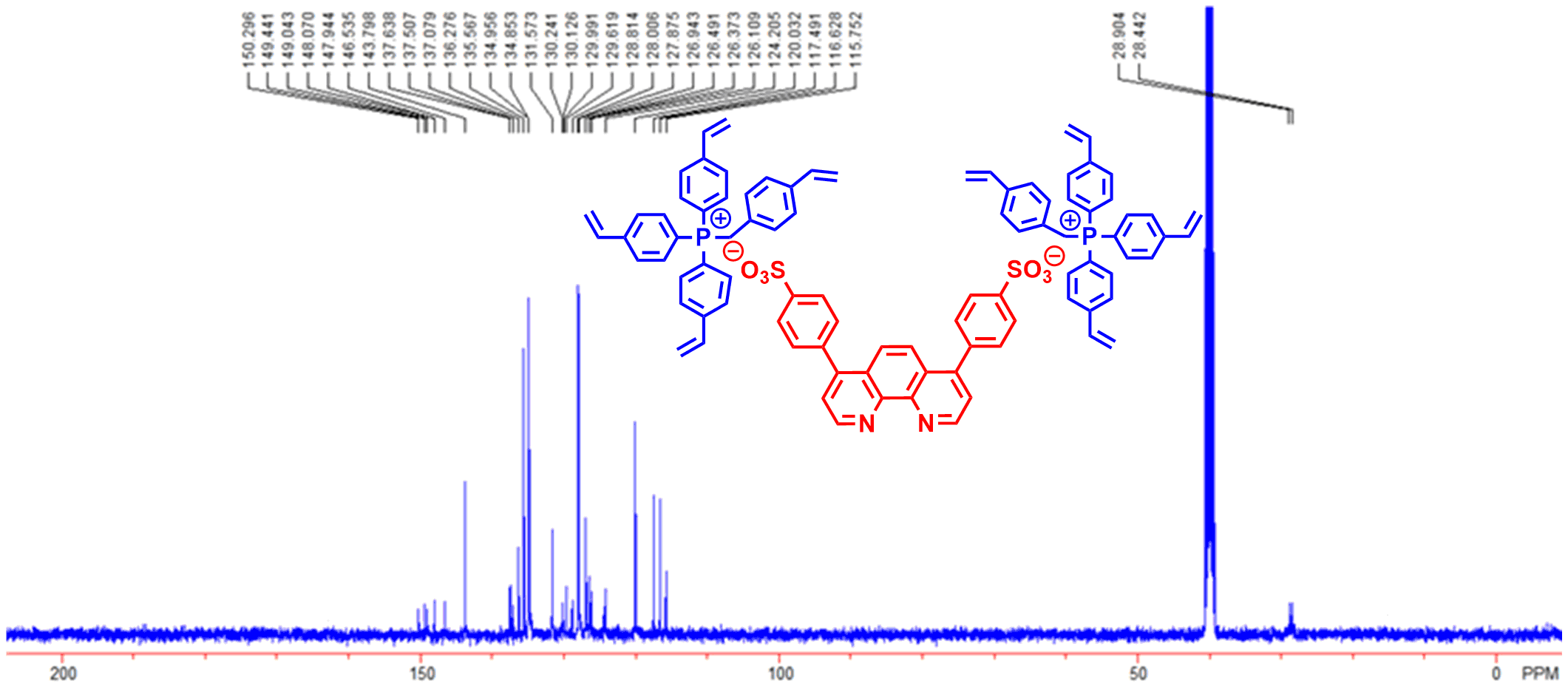
**

**Supplementary Figure 5 | ^1^H and ^13^C NMR spectra of VSA-Phen.**

**Supplementary Figure 6 | ESI-TOF-MS spectra of VSA-Phen.** Above the dashed line is the cationic part detected under the positive ion mode and below the dashed line is the anionic part detected under the negative ion mode.

**
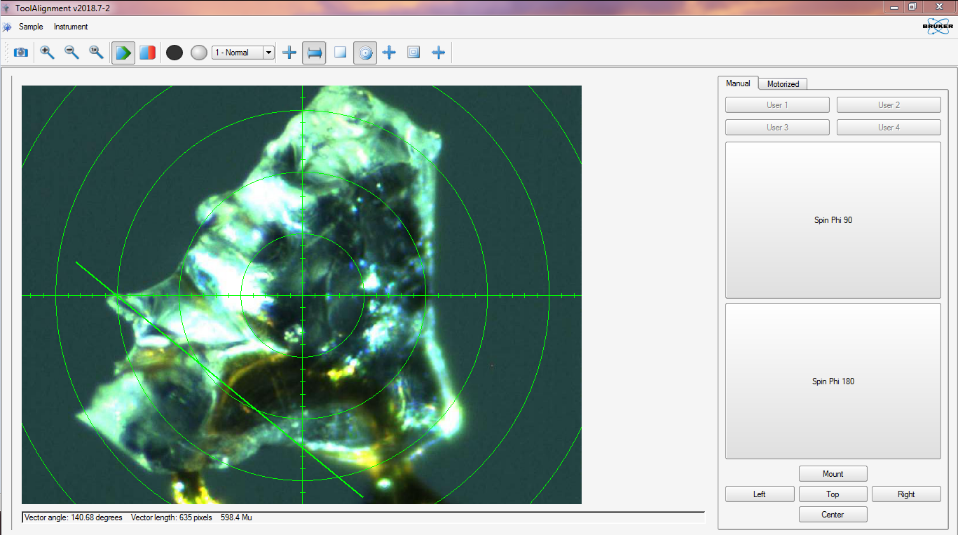
**

**Supplementary Figure 7 | Image of VSA-PPh_3_.** In contrast with the cationic template and sulfonic salts, in which single crystals can be readily obtained, the resulting supramolecular assemblies were glass-like amorphous solids, and any attempts to grow crystals were unsuccessful.




**Supplementary Figure 8 |** **Solid-state ^13^C NMR spectrum of PSA-PPh_3_.** Peak d also includes unpolymerized vinyl groups.

**Supplementary Figure 9 | Pore size distribution calculated based upon nonlocal density functional theory (NLDFT) of PSA-PPh_3_.**

**Supplementary Figure 10 |** **PXRD pattern of PSA-PPh_3_.**

**Supplementary Figure 11 | TG curve of PSA-PPh_3_.**

**
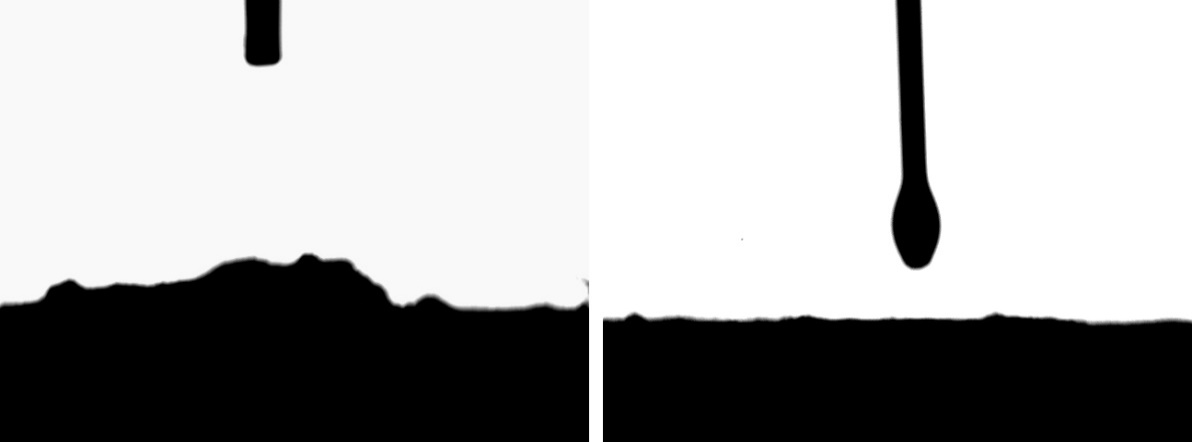
**

**Supplementary Figure 12 | Contact angel tests.** Photographs of a water (left) and 1-octene (right) droplet on the surface of PSA-PPh_3_. PSA-PPh_3_ displays excellent amphiphilicity, given the fact that when a water or 1-octene droplet contacts on its surface, both of them can be quickly absorbed, giving rise to a swelled surface.

Supplementary Figure 13 | Vapor sorption tests. Water and toluene adsorption (solid symbols) and desorption (open symbols) isotherms of PSA-PPh_3_ collected at 298 K.

Supplementary Figure 14 | Particle size distribution analysis. DLS analysis in water of PSA/PPh_3_.

**Supplementary Figure 15 | Porosity evaluation.** N_2_ sorption isotherms of Rh/PSA-PPh_3_ collected at 77 K. The BET surface area was calculated to be 536 m^2^ g^-1^.





**
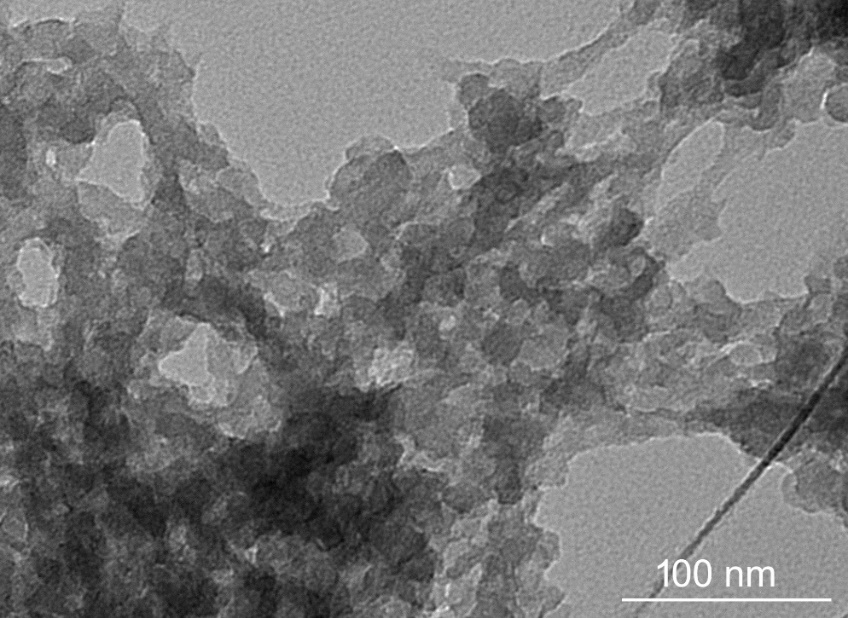
**

**Supplementary Figure 16 | SEM (top) and TEM (bottom) images of Rh/PSA-PPh_3_.**

**
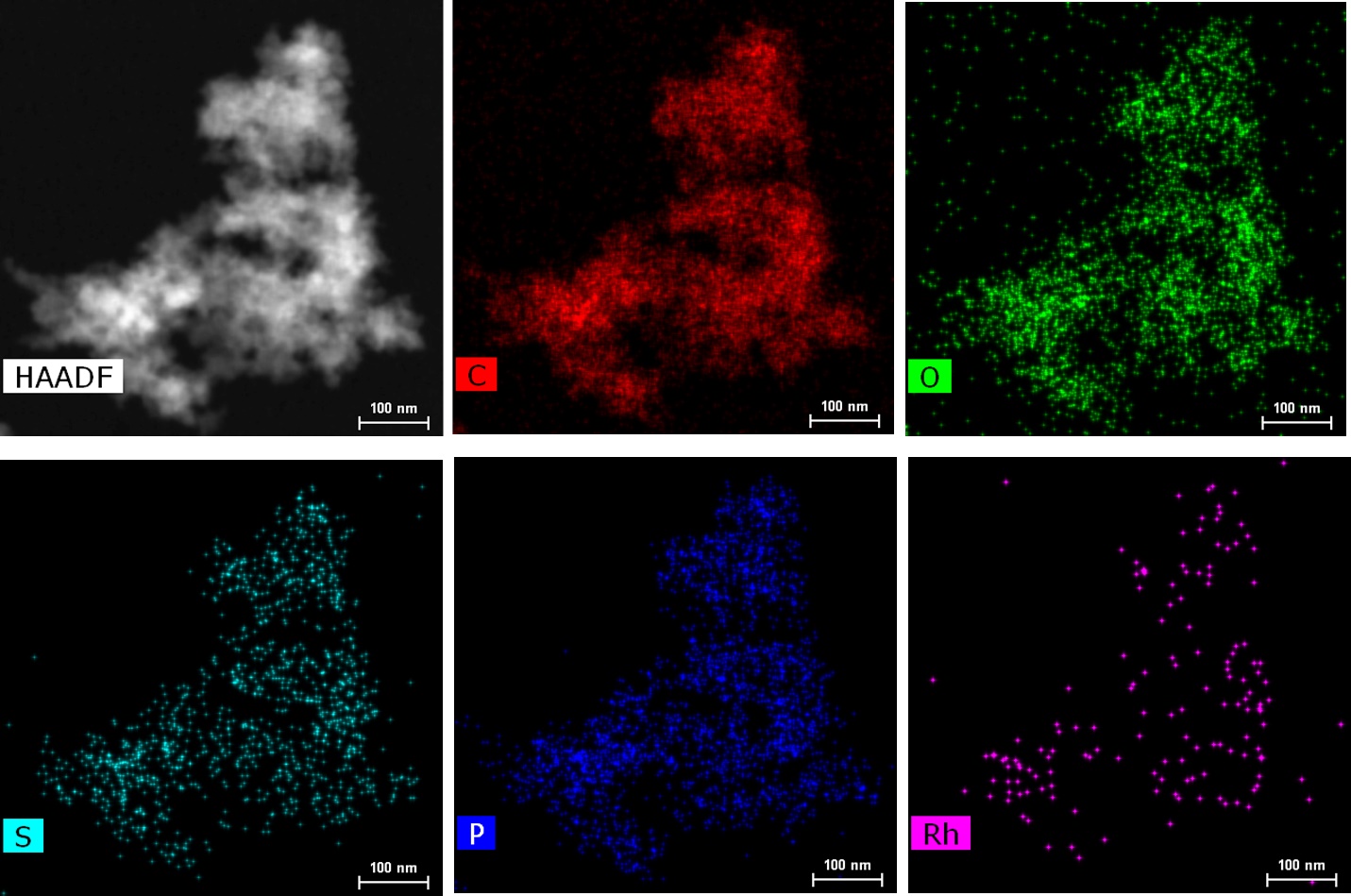
**

**Supplementary Figure 17 |** **HAADF-STEM and corresponding elemental mapping of Rh/PSA-PPh_3_.**

**Supplementary Figure 18 |** **Rh3*d* and P2*p* XPS spectra.** The two distinct signals at 132.8 eV and 130.9 eV in the P2*p* spectrum of PSA-PPh_3_ are ascribed to the phosphonium species from the template and phosphine species from the PPh_3_ moieties, respectively.

**Supplementary Figure 19 |** **IR spectra of Rh(CO)_2_(acac), PSA-PPh_3_, and Rh/PSA-PPh_3_.**

**Supplementary Figure 20 |** Dependences of catalytic performance in the hydroformylation of 1-octene on the phosphine/Rh ratios in xRh/PSA-PPh_3_, wherein x is the ratio of PPh_3_ moieties to Rh. Reaction conditions: 1-octene (561 mg, 5 mmol), xRh/PSA-PPh_3_ (0.00167 mmol of Rh species), 100 °C, CO/H_2_ = 1:1 (2.0 MPa), and 1 h.

**

**

**Supplementary Figure 21 | Solid-state ^13^C NMR spectra of PSA-Xantphos.** Peak e also includes unpolymerized vinyl groups.

**Supplementary Figure 22 | Solid-state ^31^P NMR spectra of PSA-Xantphos.** The ^31^P MAS NMR spectrum of PSA-Xantphos gave two distinctive signals at 21.8 and -15.7 ppm, attributable to the phosphonium species from the template and phosphine species from the Xantphos moieties, respectively.

**
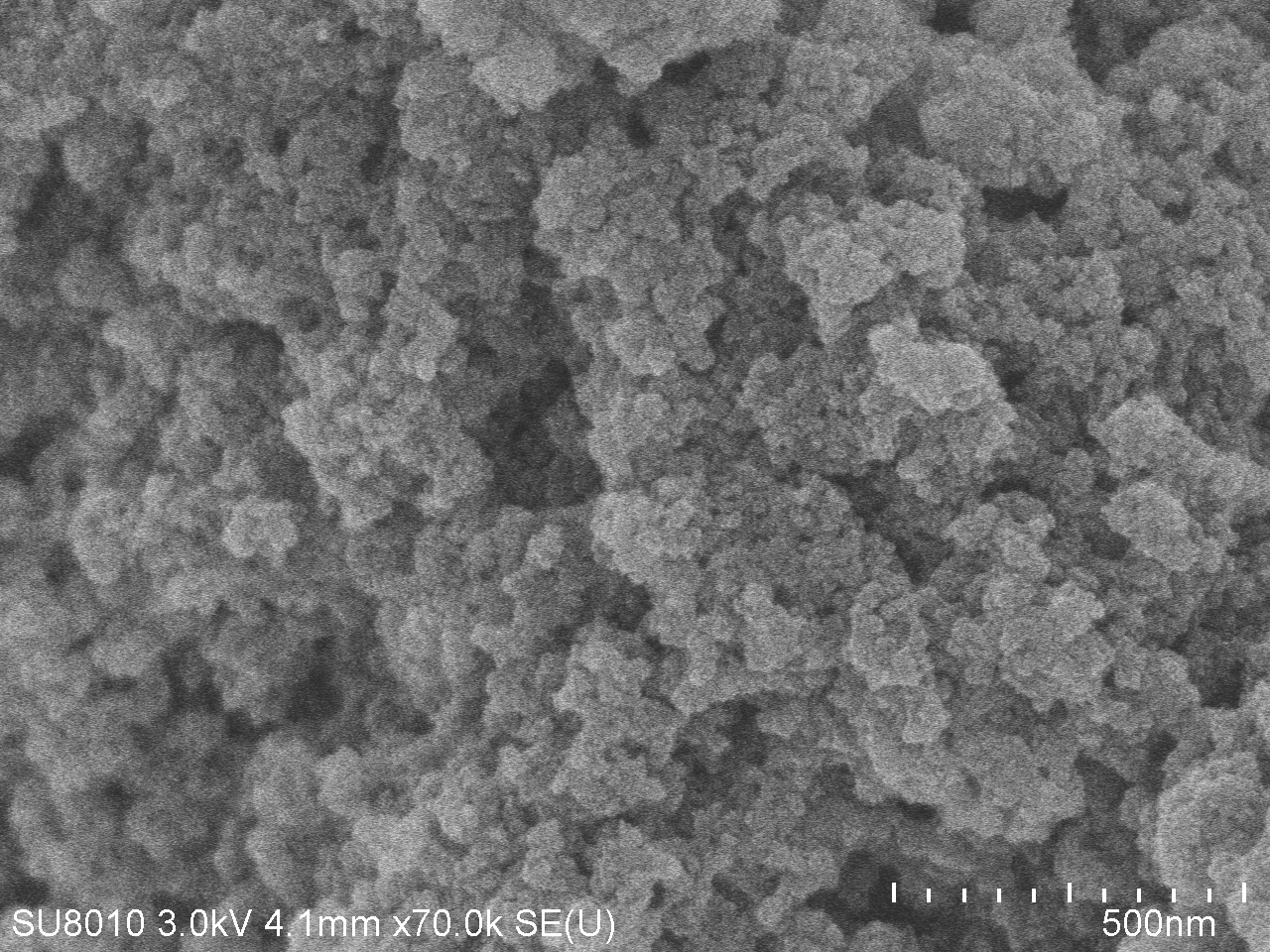
**


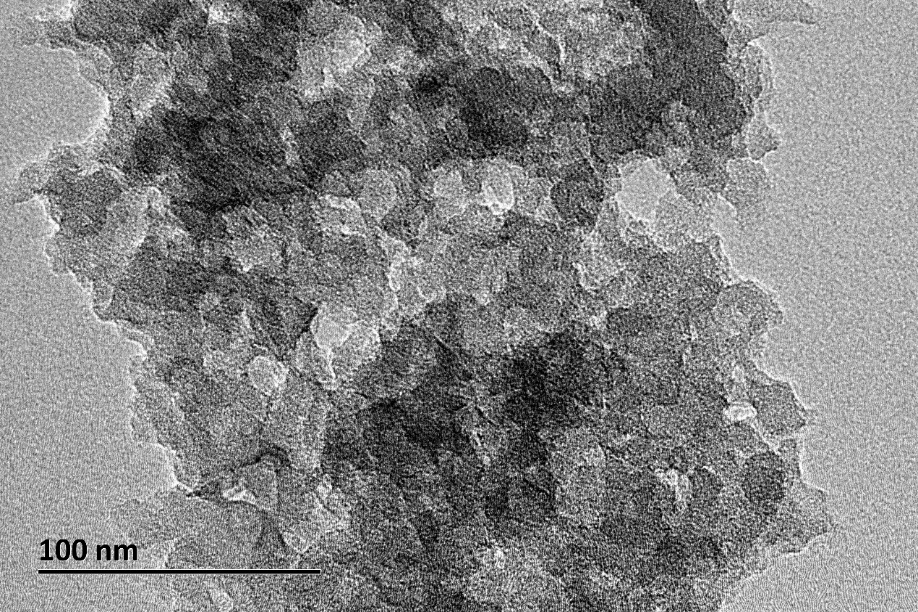


**Supplementary Figure 23 |** **SEM (top) and TEM (bottom) images of PSA-Xantphos.**

**Supplementary Figure 24 |** **N_2_ sorption isotherms and corresponding DFT pore size distribution of PSA-Xantphos.** The BET surface area was calculated to be 290 m^2^ g^-1^.

**Supplementary Figure 25 | PXRD pattern of PSA-Xantphos.**

**Supplementary Figure 26 |** **TG curve of PSA-Xantphos.**

**
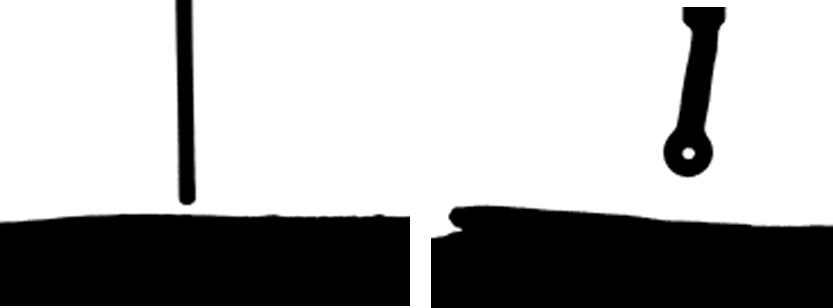
**

**Supplementary Figure 27 | Contact angle tests.** Photographs of a water (left) and 1-octene (right) droplet on the surface of PSA-Xantphos. PSA-Xantphos displays excellent amphiphilicity, given the fact that when a water or 1-octene droplet contacts on its surface, both of them can be quickly absorbed, giving rise to a swelled surface.

**
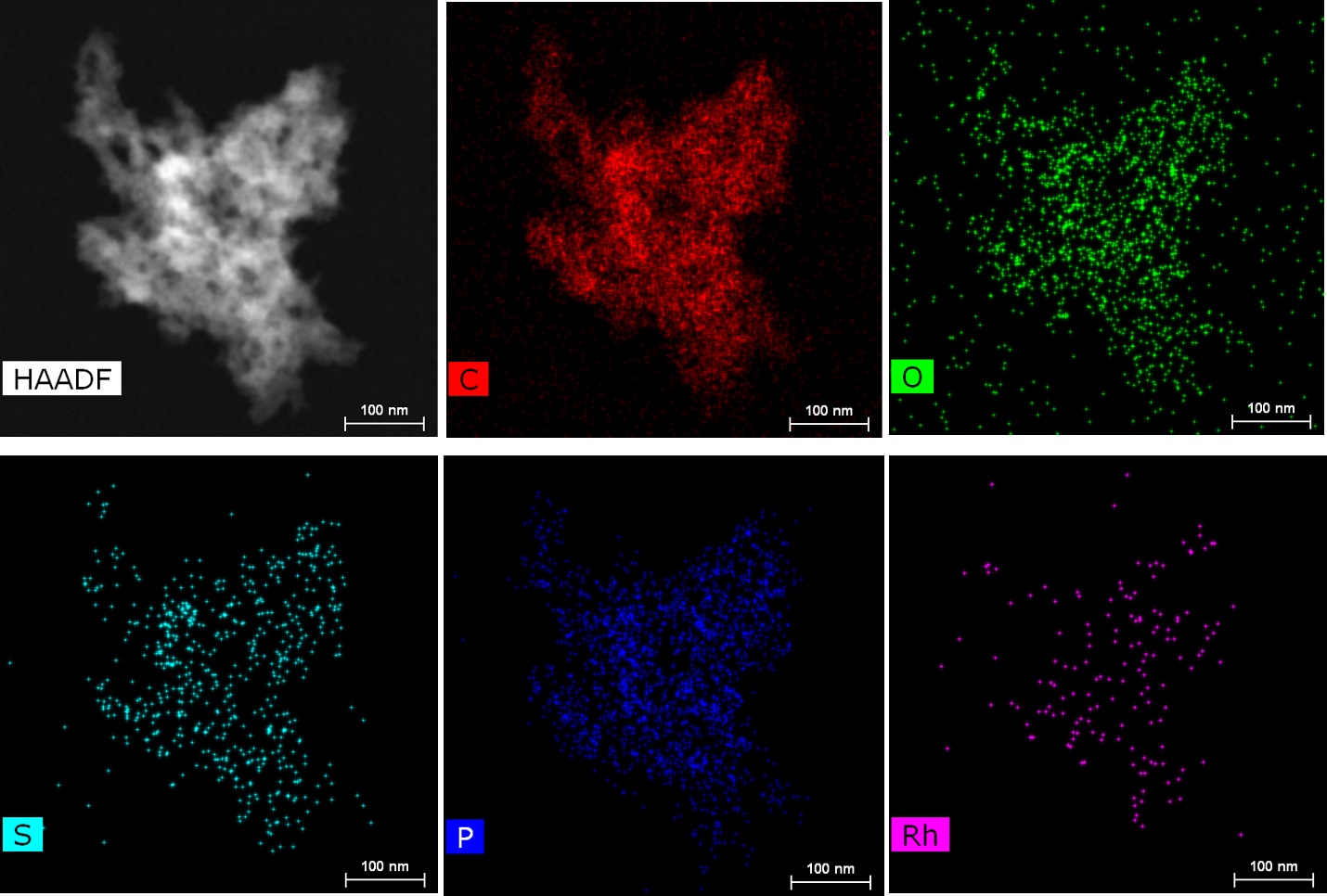
**

**Supplementary Figure 28 |** **HAADF-STEM and corresponding elemental mapping of Rh/PSA-Xantphos, wherein the P/Rh ratio is 10.**

**Supplementary Figure 29 |** **Rh3*d* and P2*p* XPS spectra.** The two distinct signals at 132.6 eV and 130.9 eV in the P2*p* spectrum of PSA-Xantphos are ascribed to the phosphonium species from the template and phosphine species from the Xantphos moieties, respectively. The XPS spectra of Rh/PSA-Xantphos demonstrated the binding energies of Rh3*d*_5/2_and Rh3*d*_3/2_ at 308.7 and 313.4 eV, respectively, which are lower than those of Rh(CO)_2_(acac) (309.3 and 314.0 eV). Meanwhile, the P2*p* binding energy of the phosphine species in Rh/PSA-Xantphos (131.3 eV) is higher than that of the parent PSA-Xantphos (130.9 eV). These results suggest that strong interactions exist between the Rh species and Xantphos moieties in PSA-Xantphos.


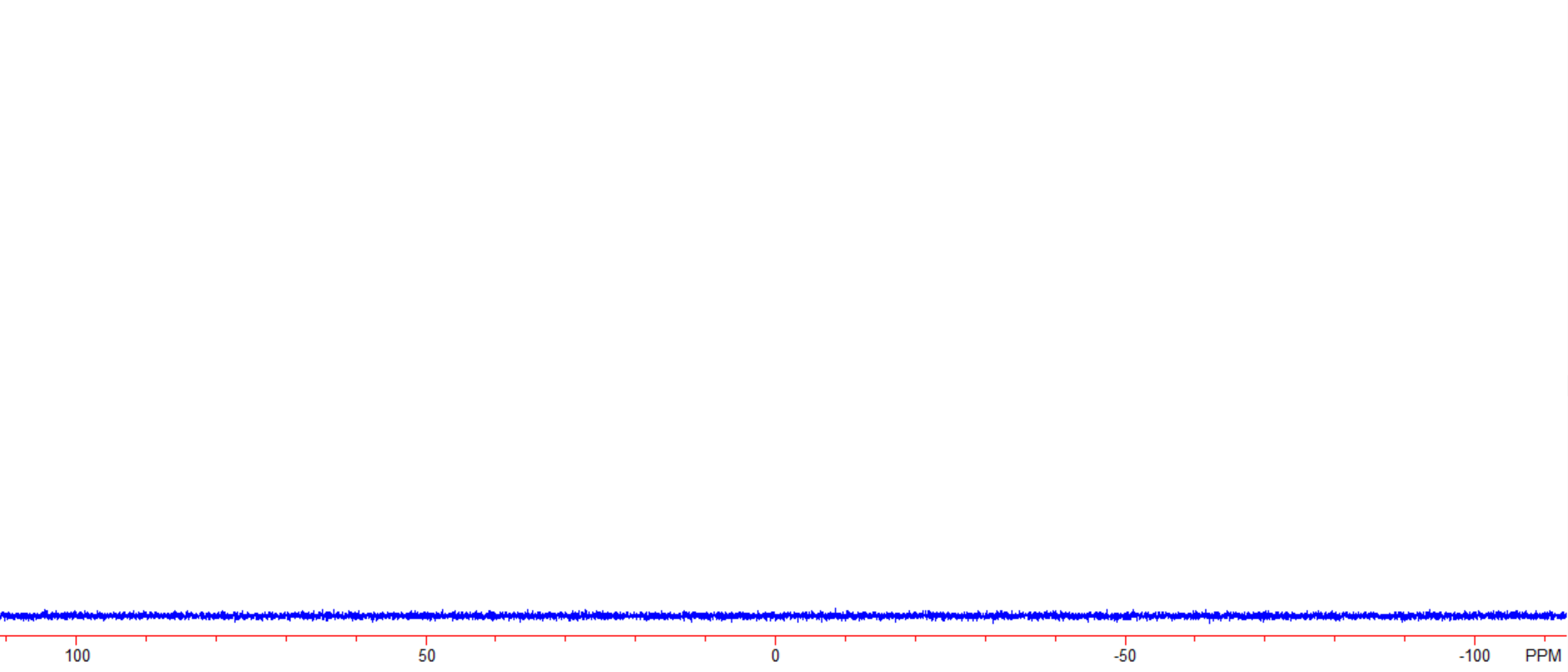


**Supplementary Figure 30 | ^31^P NMR spectrum of the filtrate after the reaction catalyzed by Rh/PSA-Xantphos.** Given that there are no detectable P species, indicative of no leached Xantphos species.


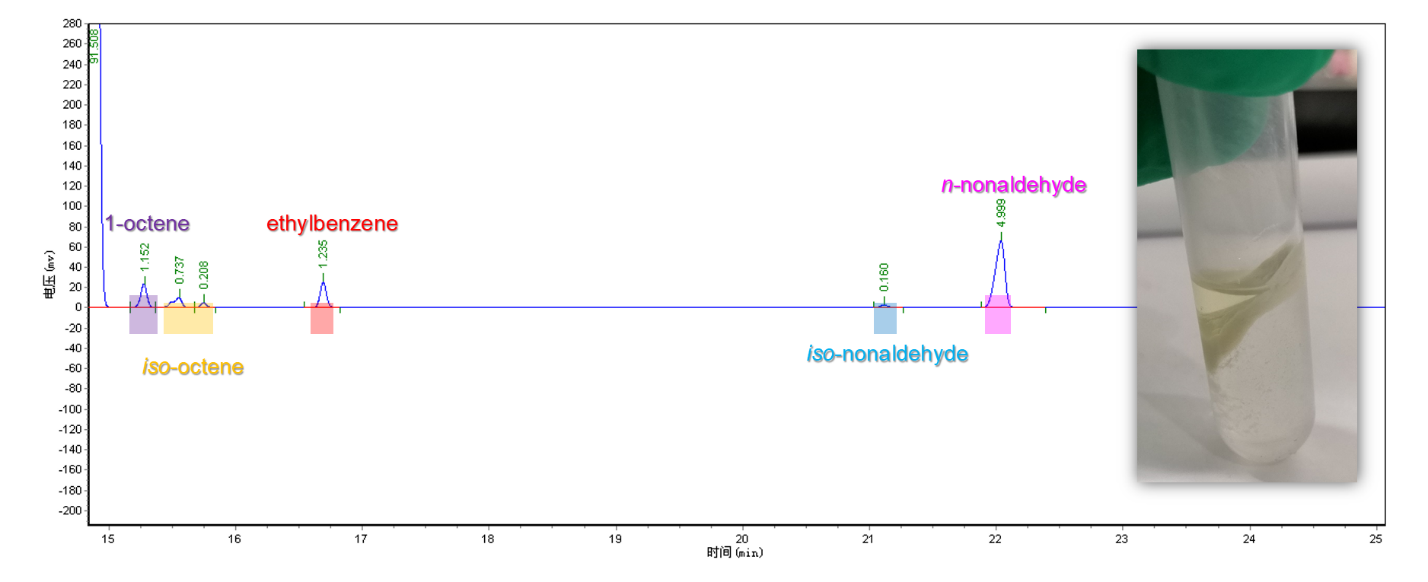


**Supplementary Figure 31 | Product analysis.** Gas chromatography spectrum of the organic phase composition after the hydroformylation of 1-octene catalyzed by Rh/PSA-Xantphos, inset: photograph of the distribution of organic compounds, catalyst, and water after the reaction.


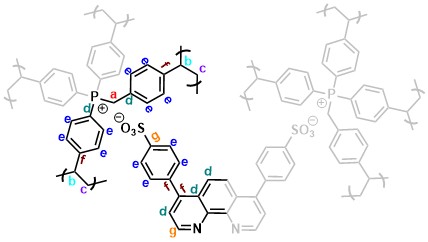

**Supplementary Figure 32 |** **Solid-state ^13^C NMR spectrum of PSA-Phen.** Peak d also includes unpolymerized vinyl groups.

**Supplementary Figure 33 | Solid-state ^31^P NMR spectra of PSA-Phen.** The ^31^P MAS NMR spectrum of PSA-Phen gave only one signal at 20.5, attributable to the phosphonium species from the template.

**Supplementary Figure 34 |** **N_2_ sorption isotherms and corresponding pore size distribution of PSA-Phen.** The BET surface area was calculated to be 389 m^2^ g^-1^.

**
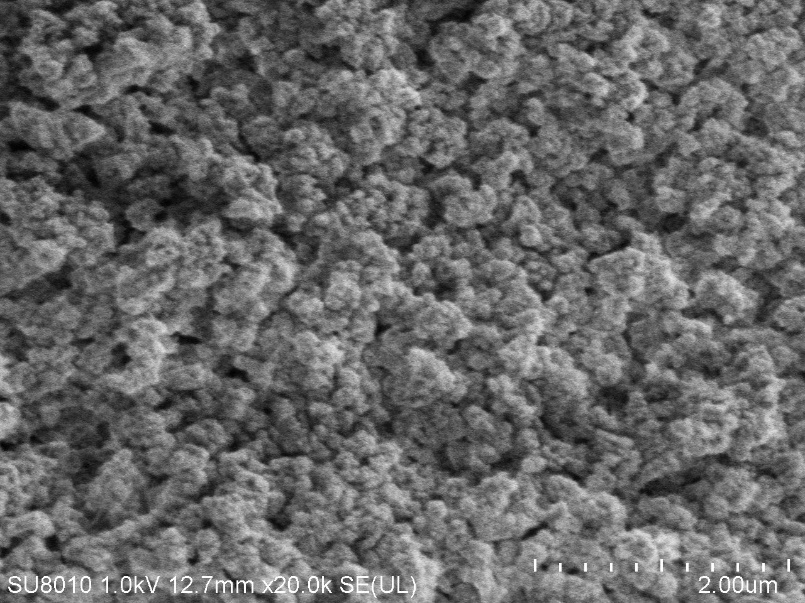
**

**
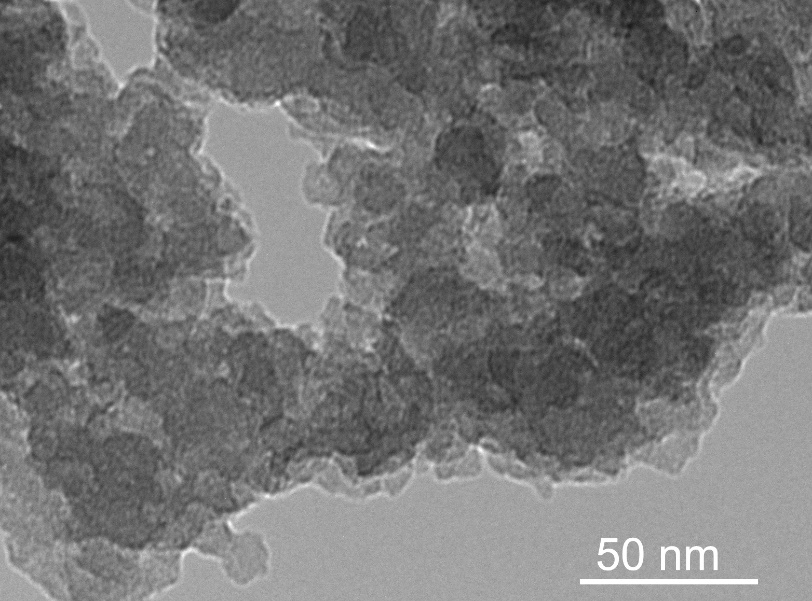
**

**Supplementary Figure 35 |** **SEM (top) and TEM (bottom) images of PSA-Phen.**

**Supplementary Tables**

**Supplementary Table 1 | Recycling tests of Rh/PSA-Xantphos in the hydroformylation of 1-octene. *^a^***

| Run | Aldehyde productivity (mmol h^-1^) |
| --- | --- |
| 0 | 0.59 |
| 1 | 0.58 |
| 2 | 0.59 |
| 3 | 0.61 |
| 4 | 0.57 |
| 5 | 0.58 |

*^a^* Reaction conditions: 1-octene (0.28 g, 2.5 mmol), Rh/PSA-Xantphos (P/Rh = 10, 54.8 mg with 0.00167 mmol of Rh species), 120 °C, CO/H_2_ = 1:1 (2.0 MPa), and 2 h. After completion of the first run, fresh starting material (1-octene, 2.5 mmol) was added directly to the reaction system, and the reaction was monitored as before.

**Supplementary Table 2 | The effect of water on the catalytic performance of Rh/PSA-PPh_3_ in the hydroformylation of 1-octene. *^a^***

| Entry | Water (mL) | Aldehyde productivity  (mmol h^-1^) | Aldehyde selectivity (%) |
| --- | --- | --- | --- |
| 1 | 0 | 0.55 | 70.0 |
| 2 | 0.1 | 0.79 | 85.7 |
| 3 | 0.3 | 0.88 | 95.3 |
| 4 | 1.0 | 1.84 | 96.1 |
| 5 | 10 | 1.93 | 96.5 |
| 6 | 40 | 1.54 | 96.6 |

*^a^* Reaction conditions: 1-octene (2.24 g, 20 mmol), Rh/PSA-PPh_3_ (35.8 mg with 0.00167 mmol of Rh species), 100 °C, CO/H_2_ = 1:1 (2.0 MPa), and 1 h.

**Supplementary Table 3 | The catalytic performance of Rh/PSA-PPh_3_ in the hydroformylation of 1-octene under various reaction meida. *^a^***

| Entry | Solvent (mL) | pH | Conv.(%) | Select.(%) |
| --- | --- | --- | --- | --- |
| 1 | H_2_O (10) | 7 | 47.3 | 98.4 |
| 2 | H_2_O (10) | 3 | 38.0 | 95.5 |
| 3 | H_2_O (10) | 11 | 15.3 | 96.5 |
| 4*^b^* | H_2_O (10) | 7 | 96.3 | 98.4 |
| 5*^b^* | toluene (10) | -- | 55.1 | 76.3 |
| 6*^b^* | toluene/H_2_O (5/5) | -- | 77.8 | 82.3 |

*^a^* Reaction conditions: 1-octene (561 mg, 5 mmol), Rh/PSA-PPh_3_ (35.8 mg with 0.00167 mmol of Rh species), 100 °C, CO/H_2_ = 1:1 (2.0 MPa) and 1 h. *^b^* Reaction for 4 h.

**Supplementary Table 4 | The catalytic performance of Rh/PSA-PPh_3_ in the hydroformylation of 1-octene under various conditions. *^a^***

| Entry | Stirring speed (rpm) | Catalyst amount (mg) | P(syngas) (MPa) | Conv.(%) | Select.(%) |
| --- | --- | --- | --- | --- | --- |
| 1 | 600 | 35.8 | 2 | 47.3 | 98.4 |
| 2 | 300 | 35.8 | 2 | 23.4 | 98.2 |
| 3 | 900 | 35.8 | 2 | 47.1 | 98.4 |
| 4 | 600 | 53.7 | 2 | 51.6 | 98.5 |
| 5 | 600 | 17.9 | 2 | 27.6 | 98.4 |
| 6 | 600 | 35.8 | 1 | 15.8 | 85.5 |
| 7 | 600 | 35.8 | 3 | 50.6 | 98.5 |

*^a^* Reaction conditions: 1-octene (561 mg, 5 mmol), H_2_O (10 mL), 100 °C, and 1 h. These data are a clear indication of mass transfer limited by the availability of gaseous reagent.

**Supplementary Table 5 | Performance of representative catalytic systems in the literature of hydroformylation of 1-octene using water as a medium.**

| Catalyst | TOF (h^-1^) | Aldehyde select. (%) | Ref |
| --- | --- | --- | --- |
| Rh/PSA-PPh_3_ | 1158 | 96.5 | This work |
| Rh/tppts & Styrene lattices | 32.1 | 90 | a |
| Rh/tppts & Αcd/PEG | 35.5 | 95 | b |
| Rh-PEVV | 55.7 | 44 | c |
| RhPPA(Na^+^)/DPPEA | 25 | 60 | d |
| Rh/Amphiphilic diphosphines | 21 | 97.6 | e |
| Rh/tppts-SAP catalyst | 15 | 84 | f |
| Rh/TPPTS-DMCD | 15.6 | 75 | g |
| Rh/polyethylene glycolate | 609 | -- | h |
| Rh(μ-OMe)(cod)_2_/dppbts | 6.3 | 31 | i |
| Rh/phosphinoimidazolium salts | 960 | 98 | j |
| MCM-C18N-1.8 | 392 | 79 | k |
| Rh/TPPTS-[OctMim]Cl | 650 | 81 | l |
| Rh/TPPTS[OctMinBr] | 1150 | 91.5 | m |

Note: TOF values were calculated based on the following equation: aldehyde yield (mol)/[Rh (mmol) x time (h)]

*^a^* Kunna, K., Müller, C., Loos, J. & Vogt, D. Aqueous-phase hydroformylation of 1-octene:

styrene latices as phase-transfer agents. *Angew. Chem. Int. Ed.* **45**, 7289-7292 (2006).

*^b^* Potier, J., Menuel, S., Chambrier, M.-H., Burylo, L., Blach, J.-F., Woisel, P., Monflier, E. & Hapiot, F. Pickering emulsions based on supramolecular hydrogels: application to higher olefins’ hydroformylation. *ACS Catal.* **3**, 1618-1621 (2013).

*^c^* Chen, J. & Alper, H. A novel water-soluble Rhodium-poly(enolate-co-vinyl alcohol-co-vinyl acetate) catalyst for the hydroformylation of olefins. *J. Am. Chem. Soc.* **119**, 893-895 (1997).

*^d^* Nait, A. & Alper, H. A new, efficient, and in some cases highly regioselective water-soluble polymer Rhodium catalyst for olefin hydroformylation. *J. Am. Chem. Soc.* **120**, 1466-1468 (1998).

*^e^* Goedheijt, S. M., Hanson, B. E., Reek, J. N. H., Kamer, P. C. J. & van Leeuwen, P. W. N. M. Accelerated biphasic hydroformylation by vesicle formation of amphiphilic diphosphines. *J. Am. Chem. Soc.* **122**, 1650-1657 (2000).

*^f^* Horváth, I. T. Hydroformylation of olefins with the water soluble HRh(CO)[P(m-C_6_H_4_SO_3_Na)_3_]_3_ in supported aqueous-phase. Is it really aqueous. *Catal. Lett.* **6**, 43-48 (1990).

*^g^* Shimizu, S., Shirakawa, S., Sasaki, Y. & Hirai, C. Novel water-soluable Calix[4]arene ligands with phosphane-containing groups for dual functional metal-complex catalysts: the biphasic hydroformylation of water-insoluble olefins. *Angew. Chem. Int. Ed.* **39**, 1256-1259 (2000).

*^h^* Borrmann, T., Roesky, H. W. & Ritter, U. Biphasic hydroformylation of olefins using a novel water soluble rhodium polyethylene glycolate catalyst. *J. Mol. Catal. A* **153**, 31-48 (2000).

*^i^* Giménez-Pedrós, M., Aghmiz, A., Claver, C., Masdeu-Bultó, A. M., Sinou, D. Micellar effect in hydroformylation of high olefin catalysed by water-soluble rhodium complexes associated with sulfonated diphosphines. *J. Mol. Catal. A* **200**, 157-163 (2003).

*^j^* Chen, S.-J., Wang, Y.-Y., Li, Y.-Q., Zhao, X.-L. & Liu, Y. Ion-pair effect of phosphino-imidazolium salts on Rh(III)-complex catalyzed hydroformylation of 1-octene in water suspension. *Catal. Commun.* **50**, 5-8 (2014).

*^k^* Zhao, Y., Zhang, X., Sanjeevi, J. & Yang, Q. Hydroformylation of 1-octene in Pickering emulsion constructed by amphiphilic mesoporous silica nanoparticles. *J. Catal.* **334**, 52-59 (2016).

*^l^* Desset, S. L., Reader, S. W. & Cole-Hamilton, D. J. Aqueous-biphasic hydroformylation of alkenes promoted by “weak” surfactants. *Green. Chem.* **11**, 630-637 (2009).

*^m^* Desset, S. L., Cole-Hamilton, D. J. & Foster, D. F. Aqueous-biphasic hydroformylation of higher alkenes promoted by alkylimidazolium salts. *Chem. Commun.* 1933-1935 (2007).

**Supplementary Methods**

***Chemicals***

Solvents were purified according to standard laboratory methods. Other commercially available reagents were purchased in high purity and used without further purification.

***Material synthesis***

***Synthesis of vinyl-functionalized cationic template (4-vinyl-benzyl)-tris-(4-vinyl-phenyl)-phosphonium chloride***

***

***

*tris-(4-vinylphenyl)-phosphine (1):* First, (4-vinylphenyl)magnesium bromide was prepared as follows: after the addition of 4-bromostyrene (18.3g, 100 mmol) to Mg powder 2.88 g, 120 mmol) in THF (150 mL) at 0 ºC under N_2_, the mixture was further stirred at 0 ºC for one hour. Next, PCl_3_ (4.5 g in 30 mL THF) was added to the solution over 30 min at 0 °C, followed by stirring at room temperature overnight. After quenching by the addition of 50 mL of saturated NH_4_Cl aqueous solution, the mixture was extracted with Et_2_O. The organic layer was washed with brine, dried over MgSO_4_, and concentrated under vacuum. The residue was purified by column chromatography over silica gel (EtOAc:hexane = 1:10) to give **1** as a white solid. ^1^H NMR (400 MHz, CDCl_3_, 298K, TMS): δ 7.37 (d, 6H), 7.22-7.31 (t, 6H), 6.64-6.75 (t, 3H), 5.75(d, 3H), 5.27 (d, 3H) ppm. ^13^C NMR (100 MHz, CDCl_3_): δ 114.7, 126.28, 126.35, 133.78, 133.97, 136.35, 136.53, 136.63, 138.00 ppm. ^31^P NMR (162 MHz): δ -6.72 ppm.

*(4-vinyl-benzyl)-tris-(4-vinyl-phenyl)-phosphonium chloride (V-QP) (2):* 4-Vinylbenzyl chloride (0.76 g, 5 mmol) was added to a solution of tris-(4-vinylphenyl)-phosphine (1.31 g, 5 mmol) and acetone (10 mL). The mixture was heated to 60 °C for 72 h under N_2_ atmosphere, then cooled to room temperature. A solid product was precipitated out in ether and was then filtered and dissolved in the minimum amount of ethanol. This process was repeated twice and the final product as a white solid was obtained by evaporation under reduced pressure. ^1^H NMR (400 MHz, CDCl_3_, 298K, TMS): δ 7.66-7.78 (m, 6H), 7.52-7.62 (m, 6H), 7.08-7.18 (m, 4H), 6.67-6.79 (t, 3H), 6.53-6.65 (t, 1H), 5.92 (d, 3H), 5.66(d, 1H), 5.50 (d, 2H), 5.49 (d, 3H), 5.22 (d, 1H). ^13^C NMR (100 MHz, CDCl_3_): δ 30.25, 30.72, 114.56, 116.16, 117.03, 119.00, 126.45, 126.48, 127.01, 127.10, 127.48, 127.61, 131.81, 131.86, 134.64, 134.74, 135.05, 136.10, 137.34, 143.74 ppm. ^31^P NMR (162 MHz): δ 21.98 ppm.

***Synthesis of vinyl-functionalized ion pair-directed supramolecular assemblies (VSA)***

*

*

*VSA-PPh_3_.* The cationic template (V-QP, 500 mg) was dissolved in DMF (5 mL), followed by the addition of tppts (**3**, 228 mg). After being stirred at room temperature under N_2_ atmosphere for 2 h, the formed precipitate (NaCl) was removed by centrifugation and the resulting supernatant was poured into 200 mL of ether. The title product was precipitated out during this process, which was collected by filtration, washing with ether, and drying under vacuum at room temperature, yielding a yellowish solid. ^1^H NMR (400 MHz, CDCl_3_, 298K, TMS): δ 7.90-7.98 (m, 3H), 7.80-7.87 (m, 18H), 7.55-7.69 (m, 24H), 7.40-7.51 (m, 3H), 7.34-7.39 (m, 6H), 7.26-7.34 (m, 3H), 7.01-7.09 (m, 3H), 6.94-7.01 (m, 6H), 6.81-6.93 (t, 9H), 6.60-6.72 (m, 3H), 6.08-6.20 (d, 9H), 5.78-5.88 (d, 3H), 5.50-5.61 (d, 9H), 5.24-5.31 (d, 3H), 5.06-5.16 (d, 6H) ppm. ^31^P NMR (162 MHz): δ -5.17, 21.80 ppm. ESI-TOF (m/z): Calcd. [C33H30ClP-Cl]^+^ for: 457.208, found: 457.207. Calcd. [C18H12Na3O9PS3-2Na]^2-^ for: 249.72, found: 249.97. Calcd. [C18H12Na3O9PS3-3Na]^3-^ for: 166.48, found: 166.31.

*VSA-Xantphos.* SA-Xantphos was synthesized following a similar procedure as that of SA-PPh_3_ except that the sodium salt of sulfonated Xantphos (**4**, 397 mg) was used instead of tppts, which was obtained as a yellowish solid. ^1^H NMR (400 MHz, CDCl_3_, 298K, TMS): δ 7.80-7.87 (m, 12H), 7.71-7.75 (d, 2H), 7.60-7.68 (m, 12H), 7.35-7.40 (m, 4H), 7.31-7.34 (m, 12H), 7.05-7.12 (m, 10H), 6.94-7.00 (m, 4H), 6.84-6.90 (t, 6H), 6.62-6.70 (t, 2H), 6.10-6.18 (d, 6H), 5.79-5.86 (d, 2H), 5.53-5.60 (d, 6H), 5.24-5.30 (d, 2H), 5.08-5.15 (d, 4H), 1.55 (s, 6H) ppm. ^31^P NMR (162 MHz): δ -18.17, 21.78 ppm. ESI-TOF (m/z): Calcd. [C33H30ClP-Cl]^+^ for: 457.208, found: 457.207. Calcd. [C39H30O7P2S2-2Na]^2-^ for: 368.05, found: 368.05.

*VSA-Phen.* SA-Phen was synthesized following a similar procedure as that of SA-PPh_3_ except that the sodium salt of sulfonated 1,10-phenanthroline derivate (**5**, 273 mg) was used instead of tppts, which was obtained as a reddish solid. ^1^H NMR (400 MHz, CDCl_3_, 298K, TMS): δ 9.16 (d, 2H), 7.80-7.86 (m, 12H), 7.75-7.80 (m, 4H), 7.68-7.74 (m, 4H), 7.61-7.68 (m, 12H), 7.50-7.61 (m, 4H), 7.35 (d, 4H), 6.95-7.04 (m, 4H), 6.82-6.94 (t, 6H), 6.60-6.71 (t, 2H), 6.14 (d, 6H), 5.82 (d, 2H), 5.50-5.60 (d, 6H), 5.26 (d, 2H), 5.19 (d, 4H) ppm. ESI-TOF (m/z): Calcd. [C33H30ClP-Cl]^+^ for: 457.208, found: 457.207. Calcd. [C24H14N2O6S2-2Na]^2-^ for: 490.03, found: 490.03.

***Synthesis of porous frameworks constructed by supramolecular assemblies (PSA)***

*PSA-PPh_3_.* As a typical procedure, 500 mg of VSA-PPh_3_ was dissolved in 5 mL of DMF, followed by the addition of 25 mg of AIBN. The mixture was transferred into an autoclave at 100 ºC for 24 h. The title polymer was obtained after being washed with ethanol and evaporated under vacuum.

*PSA-Xantphos.* PSA-Xantphos was synthesized following a similar procedure as that of PSA-PPh_3_ except that 500 mg of VSA-Xantphos was used instead of VSA-PPh_3_.

*PSA-Phen.* PSA-Phen was synthesized following a similar procedure as that of PSA-PPh_3_ except that 500 mg of VSA-Phen was used instead of VSA-PPh_3_.

***Synthesis of Rh/PSA-PPh_3_ catalyst***

As a typical procedure, 0.3 g of PSA-PPh_3_, containing 0.16 mmol of PPh_3_ moieties, was swollen in 20 mL of toluene for 30 minutes, followed by the addition of 4.1 mg of Rh(CO)_2_(acac) (0.016 mmol of Rh species). After being stirred at room temperature for 12 h under N_2_ atmosphere, the mixture was filtered and washed with excess toluene, and finally dried at 50 °C under vacuum. The obtained off-white solid was denoted as Rh/PSA-PPh_3_, wherein the ligand to Rh ratio is 10. The combined toluene filtrate was evaporated under a vacuum, and aqua regia (1 mL) was added to dissolve any possible Rh species. Then, the resultant aqua regia was diluted into 10 mL for inductively coupled plasma optical emission spectroscopy (ICP-OES) tests, revealing that the Rh concentration was below the detection limit (<0.1 ppm).

Catalysts with other P/Rh ratio were synthesized according to the same procedure except that different amounts of Rh species were introduced. The ratio of P/Rh = the mole of V-SA-PPh_3_ in PSA-PPh_3_/the mole of Rh(CO)_2_/(acac).

***Synthesis of Rh/PSA-Xantphos catalyst***

As a typical procedure, 0.3 g of PSA-Xantphos, containing 0.18 mmol of Xantphos moieties, was swollen in 20 mL of toluene for 30 minutes, followed by the addition of 4.7 mg of Rh(CO)_2_(acac) (0.018 mmol of Rh species). After being stirred at room temperature for 12 h under N_2_ atmosphere, the mixture was filtrated and washed with excess toluene, and finally dried at 50 °C under vacuum. The obtained yellowish solid was denoted as Rh/PSA-Xantphos, wherein the ligand to Rh ratio is 10. The combined toluene filtrate was evaporated under a vacuum, and aqua regia (1 mL) was added to dissolve any possible Rh species. Then, the resultant aqua regia was diluted into 10 mL for inductively coupled plasma optical emission spectroscopy (ICP-OES) tests. ICP-OES results revealed that the Rh concentration was below the detection limit (<0.1 ppm).

***Calculation of the amount of 1-octene adsorbed within Rh/PSA-PPh_3_***

The absorption capability of the polymers for 1-octene from the water was evaluated as follows: 35.5 mg of Rh/PSA-PPh_3_ was introduced into a mixture of water (10 mL) and 1-octene (5 mmol, 561 mg), the same amounts as we used for catalysis. To guarantee the absorptions reached equilibrium, an overnight stirring step was used. After that, the polymer was filtered, and the absorbed 1-octene in Rh/PSA-PPh_3_ was quantified by GC using ethylbenzene as an internal standard. Whereby, the amount of the absorbed H_2_O was calculated by the following equation: the amount of absorbed H_2_O = the increased amount of Rh/PSA-PPh_3_ after absorption - the amount of absorbed 1-octene. It is shown that Rh/PSA-PPh_3_ can absorb 97.8 mg of 1-octene and 72.8 mg of water.

***Catalytic Test***

*Hydroformylation of 1-octene.* 1-octene (5 mmol), Rh catalyst, and water (10 mL) were added into a stainless steel autoclave (100 mL). After the system was sealed and purged with syngas (CO/H_2_ = 1:1) for three times, the pressure of syngas was adjusted to the desired value (2 MPa). Then the autoclave was put into a preheated oil bath with stirring (600 rpm) at 100 °C for 4 h. After the reaction, the organic phase was extracted with toluene (5 mL) and the catalyst was removed by centrifugation. The resulting supernatant was analyzed by gas chromatography using ethylbenzene as an internal standard (GC-2014C Shimadzu Co., flame ionization detector and an OV-1 capillary column).
